# Genome sequencing and comparative genomics of enterohemorrhagic *Escherichia coli* O145:H25 and O145:H28 reveal distinct evolutionary paths and marked variations in traits associated with virulence & colonization

**DOI:** 10.1186/s12866-017-1094-3

**Published:** 2017-08-22

**Authors:** Sandra C. Lorenz, Narjol Gonzalez-Escalona, Michael L. Kotewicz, Markus Fischer, Julie A. Kase

**Affiliations:** 1 U.S. Food and Drug Administration, Center for Food Safety and Applied Nutrition, Division of Microbiology, College Park, MD 20740 USA; 2 University of Hamburg, Hamburg School of Food Science, Institute of Food Chemistry, 20146 Hamburg, Germany; 3 U.S. Food and Drug Administration, Center for Food Safety and Applied Nutrition, Division of Molecular Biology, Laurel, MD 20708 USA

**Keywords:** EHEC O145:H25, Next generation sequencing, Comparative genomics, Phylogenetics, Bacterial adaptation & colonization

## Abstract

**Background:**

Enterohemorrhagic *Escherichia coli* (EHEC) O145 are among the top non-O157 serogroups associated with severe human disease worldwide. Two serotypes, O145:H25 and O145:H28 have been isolated from human patients but little information is available regarding the virulence repertoire, origin and evolutionary relatedness of O145:H25. Hence, we sequenced the complete genome of two O145:H25 strains associated with hemolytic uremic syndrome (HUS) and compared the genomes with those of previously sequenced O145:H28 and other EHEC strains.

**Results:**

The genomes of the two O145:H25 strains were 5.3 Mbp in size; slightly smaller than those of O145:H28 and other EHEC strains. Both strains contained three nearly identical plasmids and several prophages and integrative elements, many of which differed significantly in size, gene content and organization as compared to those present in O145:H28 and other EHECs. Furthermore, notable variations were observed in several fimbrial gene cluster and intimin types possessed by O145:H25 and O145:H28 indicating potential adaptation to distinct areas of host colonization. Comparative genomics further revealed that O145:H25 are genetically more similar to other non-O157 EHEC strains than to O145:H28.

**Conclusion:**

Phylogenetic analysis accompanied by comparative genomics revealed that O145:H25 and O145:H28 evolved from two separate clonal lineages and that horizontal gene transfer and gene loss played a major role in the divergence of these EHEC serotypes. The data provide further evidence that ruminants might be a possible reservoir for O145:H25 but that they might be impaired in their ability to establish a persistent colonization as compared to other EHEC strains.

**Electronic supplementary material:**

The online version of this article (doi:10.1186/s12866-017-1094-3) contains supplementary material, which is available to authorized users.

## Background

Shiga toxin-producing *Escherichia coli* (STEC) are a genetically and phenotypically extremely diverse group of *E. coli* strains characterized by the production of one or more Shiga toxins (Stx1 and Stx2). Over 250 different STEC serotypes exist of which at least 100 have been linked to human diarrhea [1, 2]. Certain STEC strains are capable of causing more severe human diseases such as hemorrhagic colitis (HC) and the life-threatening hemolytic uremic syndrome (HUS); these STEC are commonly referred to as enterohemorrhagic *E. coli* (EHEC). Besides the expression of Stx1 and/or Stx2, classical EHEC strains carry the locus of enterocyte effacement (LEE) responsible for the formation of attaching and effacing (A/E) lesions on epithelial cells, and possess a large virulence plasmid encoding enterohemolysin (EhxA) [3–5]. While EHEC O157:H7 is considered to be the most frequent cause of severe disease, non-O157 EHEC and STEC (LEE-negative STEC e.g. O104 and O113) strains are increasingly recognized as the cause of similar illnesses worldwide [1, 2, 6–9]. In fact, it is estimated that at least 50% of all STEC infections in the US are caused by non-O157 STEC/EHEC strains, many belonging to serogroups O26, O45, O103, O111, O121 and O145 also known as the “big six” [1, 9]. As a result, in addition to O157 all non-intact beef products in the US are required to be tested for the presence of these six serogroups [10].

EHEC O145 has emerged as one of the major EHEC serogroups involved in severe human disease worldwide [7, 8, 11–13]. Most clinical O145 isolates described in the literature encompass motile and nonmotile strains of serotype O145:H28 (*n* > 150) [8, 11, 13–16], while EHEC O145:H25 appear to be less frequently isolated (10 reported cases) [11, 13, 17, 18]. However, the majority of the documented EHEC O145:H25 isolates are associated with HUS (*n* = 8), emphasizing the importance of this particular serotype [11, 13, 17, 18]. But little is known about the genetic characteristics of O145:H25 or how they have evolved or were transmitted. In fact, the source of EHEC O145:H25 infections appears to be unknown, as O145:H25 have thus far only been isolated from humans. In contrast, ruminants, especially cattle have been shown to be natural reservoirs of O145:H28 and other EHEC strains [14, 16, 19–22] and the consumption of foods contaminated with ruminant feces such as meat and milk products, fresh produce or drinking water have been identified as important transmission routes for human EHEC infections [3, 23]. It is not clear whether EHEC O145:H25 might be incapable of colonizing the bovine gut or have just failed to be isolated based on the sampling and detection methods applied.

A large number of mobile genetic elements (MGEs) have been identified in O145:H28 and other EHEC strains indicating that horizontal gene transfer (HGT) played a major role in the diversification and evolution of these pathogens [14, 24, 25]. In fact, most known and putative virulence factors are located on MGEs, such as prophages, plasmids and pathogenicity islands (PAI) and the independent acquisition of virulence factors by various serotypes contributes to the emergence of new EHEC/STEC strains [3, 14, 24–28]. Interestingly, in contrast to other non-O157 EHEC strains, EHEC O145:H28 appear to have evolved from a common ancestor with O157:H7 [14, 24]. Information regarding the evolutionary relatedness of O145:H25 and O145:H28 are sparse, but restriction fragment length polymorphisms (RFLP) analysis of the flagellar antigen-associated *fliC* genes of H25 and H28 indicated that EHEC O145:H25 and O145:H28 may belong to two different clonal lineages [13]. However, complete genome sequencing data of O145:H25 that could support this hypothesis are currently not available. Hence, in an attempt to determine the evolutionary relatedness of EHEC O145:H25 and O145:H28 as well as the genetic diversity and virulence repertoire, we sequenced the whole genome of two HUS-associated O145:H25 strains. Furthermore, in order to gain insight on the colonizing properties of O145:H25 we investigated the presence of various colonization-contributing factors (CCF) such as fimbrial and afimbrial adhesins and compared the obtained CCF profile with those found in O145:H28 and other EHEC strains.

## Results and Discussion

### General genomic features of EHEC O145:H25 strains CFSAN004176 and CFSAN004177

**Fig. 1:**
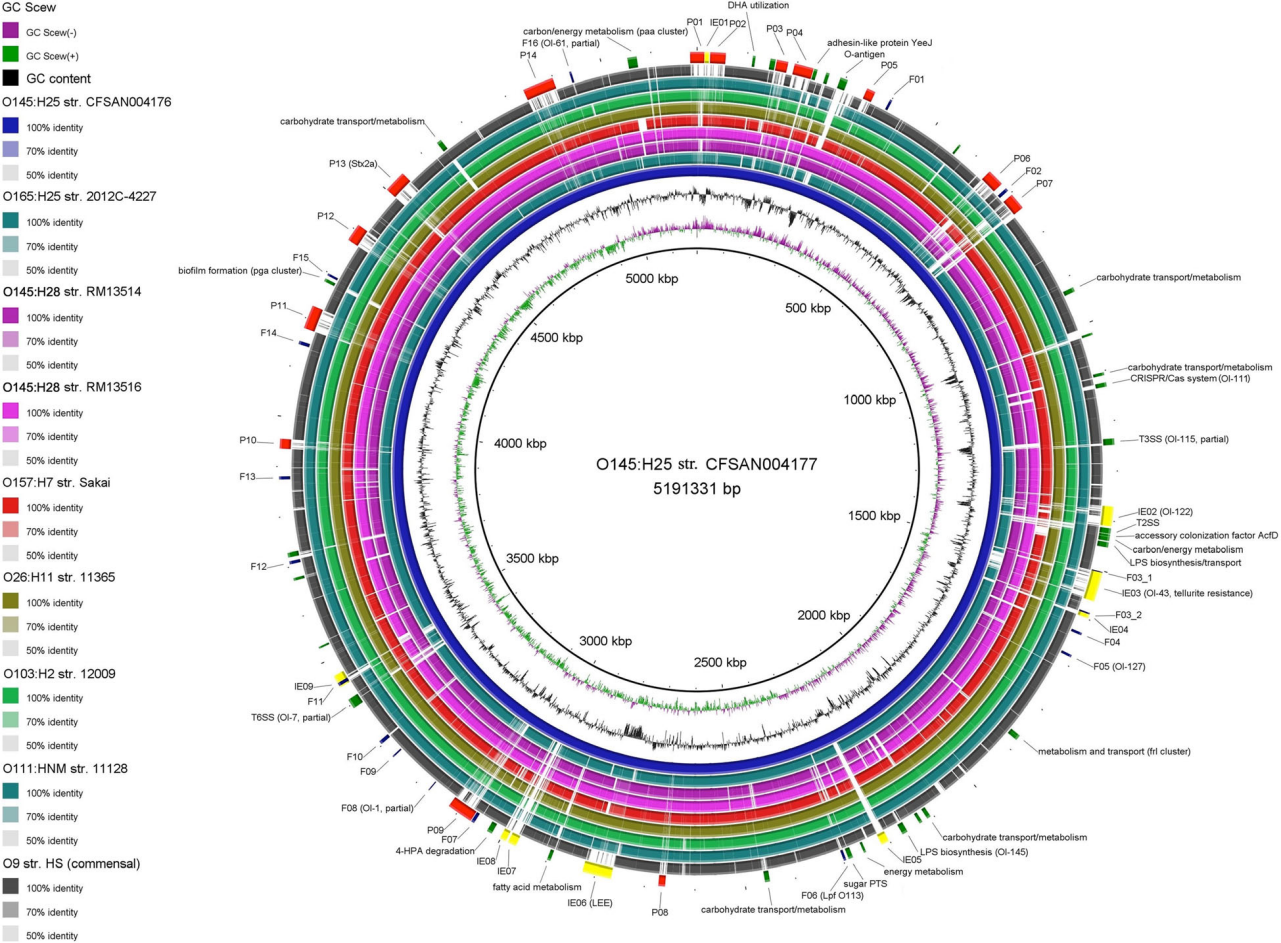
Circular map of EHEC O145:H25 strain CFSAN004177 in comparison to other EHEC strains. BLASTN comparison of the eight fully sequenced EHEC chromosomes including commensal strain HS against the chromosome of EHEC O145:H25 (CFSAN004177). Color key codes for the nine *E. coli* strains are shown on the left with nucleotide sequence identity as compared to the reference strain. The outermost ring displays prophages (*red*), IEs (*yellow*), fimbrial gene cluster (*navy*) and other genetic features (*green*) identified in the reference strain. The circular map was generated using BLAST Ring Image Generator (BRIG) with default settings

We sequenced the whole genomes of two EHEC O145:H25 strains that were isolated from HUS patients in the USA in 2003 and 2004. The chromosomes of strains CFSAN004176 and CFSAN004177 are 5193,734 bp and 5191,331 bp in size and consist of 5179 and 5193 coding DNA sequences (CDS), respectively. The chromosomal backbones of the two EHEC O145:H25 strains are nearly identical (Fig. 1); differences in chromosome size are primarily the result of variations in size and gene content of acquired MGEs (details discussed in the next sections). Furthermore, both strains belong to the same sequence type (ST) 7061, indicating their high relatedness. The two strains harbor three, nearly identical plasmids, one generally termed as pEHEC of 52,297 bp in size (pCFSAN004176_EHEC, pCFSAN004177_EHEC), one 34,714 bp plasmid (pCFSAN004176_sfp, pCFSAN004177_sfp) and one 95,721 bp/96,228 bp (CFSAN004176_PP/CFSAN004177_PP) prophage plasmid similar to Enterobacteria phage P1 (GenBank: NC_005856.1). Similar to other EHEC strains their chromosomes were found to be interrupted by various MGEs [14, 24, 25] (Fig. 1). In particular, CFSAN004176 and CFSAN004177 both carry 14 prophages, 9 integrative elements (IE) and 101 and 102 insertion sequences (IS), respectively (Table 1).

**Table 1:** General genomic characteristics of EHEC O145:H25 in comparison with O145:H28 and other EHEC genomes

Strains	O145:H25		O145:H28		O26:H11	O103:H2	O111:HNM	O157:H7	O165:H25
	CFSAN004176	CFSAN004177	RM13514	RM13516	11,365	12,009	11,128	Sakai	2012C-4227
Chromosome									
Size (kbp)	5194	5191	5586	5402	5697	5449	5371	5498	5203
GC (%)	50.5	50.5	50.7	50.7	50.7	50.7	50.6	50.5	50.7
CDSs	5179	5193	5613	5325	5780	5457	5409	5204	5032
rRNA operons	7	7	7	7	7	7	7	7	7
tRNA’s	99	96	104	98	101	98	106	105	106
Prophages (PP)	14	14	20	12	21	15	17	18	20
IEs	9	9	7	7	9	6	7	6	3
IS elements	101	102	70	61	84	76	84	60	100
ST^a^	7061	7061	32	6130	119	21	17	16	11
Plasmids									
size (kbp)	96/**52**/35	96/**52**/35	**87**/65	**98**/59	**85**/63/6/4	**76**	205/98/**78**/8/7	**93**/3	98/**75**
GC (%)	48/47/49	48/47/49	48/53	50/42	48/53/46/44	49	47/48/50/50/50	48/43	48/48
CDSs	117/58/46	120/58/46	94/69	115/73	98/93/10/3	67	222/121/72/10/10	85/3	111/81
IS elements	20	19	29	14	22	9	28	10	12
Total genome size (kbp)	5377	5374	5738	5559	5855	5525	5767	5594	5376

pEHEC-like plasmids are indicated in bold, prophage plasmids are underlined

^a^For MLST allelic profile see Additional file 4: Table S1

### Whole genome-based phylogenetic analysis among 69 fully sequenced *E. coli* strains

**Fig. 2:**
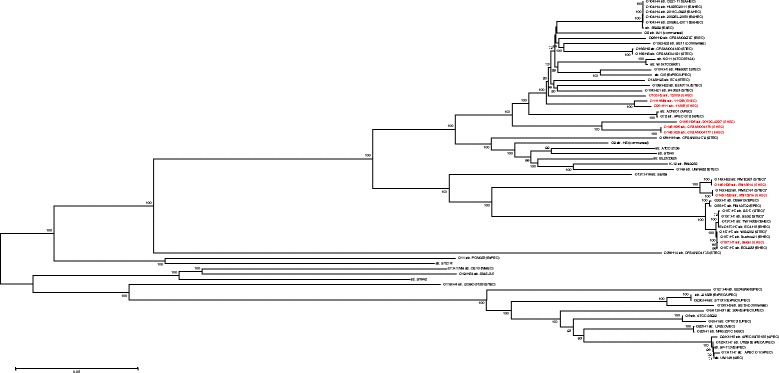
Genome-wide phylogenetic analysis of 69 fully sequenced *E. coli* strains. Whole genome wide phylogenetic tree based on SNP’s identified in 1371 core genes shared among all 69 *E. coli* strains (Additional file 2: Dataset S1 and S2). The phylogenetic analysis involved a total dataset of 86,350 SNP positions with all ambiguous positions being removed for each sequence pair. The SNP-based tree was constructed in MEGA 6 [71] using the Neighbour-joining algorithm [72] with the evolutionary history and evolutionary distance inferred using the Minimum Evolution method [73] and the Kimura 2-parameter model [74], respectively. Bootstrap values are shown on the nodes of each branch. Scale bar indicates the number of substitutions per site. EHEC strains used in this study are marked in *red*. *Carry EHEC virulence factors (*stx*, *eae* and *ehxA*) but were not clinical isolates associated with disease

To determine the evolutionary relatedness of EHEC O145:H25 and O145:H28 among 67 fully sequenced *E. coli* strains (Additional file 1: Table S1), we applied core genome multi locus sequence typing (cgMLST) followed by genome-wide SNP-based analysis of 1371 orthologous genes (Additional file 2: Dataset S1 and S2). The genome-wide SNP-based tree displayed an overall similar phylogeny of EHEC strains as previously reported [14] (Fig. 2). Furthermore, the phylogenetic analysis demonstrates that EHEC O145:H25 and O145:H28 in fact have evolved from two different clonal lineages as has been previously suspected [13]. Interestingly, while O145:H25 share a common evolutionary lineage with EHEC O26:H11 (str. 11,365), O103:H2 (str. 12,009) and O111:HNM (str. 11,128), O145:H25 appear to be most closely related to O165:H25 (str. 2012C-4227) (Fig. 2) (in the following sections referred to as O26, O103, O111 and O165). Indeed, a common evolution of O145:H25 and O165:H25 was recently assumed [29].

### Comparative genomics and *in silico* MLST analysis of O145:H25 and other EHEC strains

Based on the phylogenetic analysis and the apparently close evolutionary relationship of EHEC O145:H25 with EHEC O26, O103, O111 and O165 and EHEC O145:H28 with O157 Sakai (Fig. 2) these strains were included in the comparative genomic analysis. In order to gain insight on the genomic diversity between O145:H25 and O145:H28 as well as the other EHEC strains their genome sequences were analyzed and compared among each other. A BLASTN comparison of the nine fully sequenced EHEC strains against the chromosome of O145:H25 strain CFSAN004177 revealed a well conserved chromosomal backbone, as has been demonstrated for other EHEC strains (Fig. 1) [14, 24, 25]. However, significant differences were identified in the number and size of acquired MGEs resulting in the slightly smaller genome sizes of O145:H25 as compared to O145:H28 and other EHEC strains analyzed herein (except O165) (Table 1).

Expectedly, genomic dissimilarities were particularly observed in areas of integrated prophages and IEs (MGEs other than IS and prophages, e.g. LEE) (details discussed in next sections). However, we identified a number of non-homologous regions that did not correlate with the insertion of MGEs (here defined by the presence of an integrase gene) (Fig. 1). In particular, these regions included genes associated with LPS biosynthesis, type II secretion system (T2SS), type VI secretion system (T6SS), CRISPR (clustered regularly interspersed palindromic repeats) loci including the associated *cas* genes, and several clusters involved in metabolism and fimbrial biosynthesis (Fig. 1). Interestingly, some regions, particularly those involved in metabolism and fimbriae, were present or partially present in EHEC strains belonging to different lineages (Fig. 1, Additional file 3: Table S1). Furthermore, the flanking regions of these cluster were found to be identical in the EHEC genomes analyzed and no integrase or transposase genes were identified that could indicate recent HGT. This observation suggests that these clusters might have been integral parts of ancestral *E. coli* strains, that were retained in some EHEC lineages while being lost in others. Overall, however the majority of the identified clusters in O145:H25 was found to be absent in distantly related O145:H28 and O157 while being largely preserved in more closely related strains O26, O103, O111 and O165 including commensal *E. coli* strain HS (Fig. 1, Additional file 3: Table S1). Similarly, most of the O157-specific genomic O islands (OI) [25] were conserved in the closely related O145:H28 [14] while being absent in O145:H25 and other non-O157 EHEC strains (Additional file 3: Table S2). Hence, the data presented indicates a distinct evolutionary path for O145:H25 and O145:H28 and that in addition to HGT, gene loss appeared to have played a significant role in the diversification of these EHEC strains.

*In silico* MLST based on the seven *E. coli* housekeeping genes (*adk*, *fumC*, *gyrB*, *icd*, *purA* and *recA*) revealed that both EHEC O145:H25 strains belong to a new ST, 7061 (Table 1). Furthermore, except for *fumC* and *purA* that were identical between O145:H25 and O165, no similarities to the allelic profile of any of the other EHEC strains including O145:H28 were found (Additional file 4: Table S1).

### Plasmids

As reported previously, the EHEC strains differed significantly in the number, size, and gene contents of plasmids [14, 24]. Specifically, while the two O145:H25 strains were found to carry three plasmids, both O145:H28 strains carried two whereas the other EHEC strains analyzed herein possessed one to five plasmids (Table 1). None of the EHEC serotypes carried the same set of plasmids and in contrast to O26, O111, and O145:H28 (RM13514), no antibiotic resistance genes were identified in any of the three plasmids present in O145:H25 [14, 24]. We previously determined that the pEHEC plasmids of O145:H25 are much smaller than other pEHEC plasmids and that they lacked several pEHEC-characteristic genes (*ecf*-cluster, *espP*, *toxB*, *katP* and *stcE*) many of which were present in various combinations in O145:H28 and other EHEC strains (Table 2) [17]. The presence of several IS elements/transposase genes (Additional file 4: Table S4) and the vast genetic diversity among these pEHEC plasmids indicate a distinct evolutionary path.

**Table 2:** Identified plasmid and chromosome-encoded virulence genes and other genetic features of O145:H25 and other EHEC genomes

Strains	O145:H25		O145:H28		O26:H11	O103:H2	O111:HNM	O157:H7	O165:H25
	CFSAN004176	CFSAN004177	RM13514	RM13516	11,365	12,009	11,128	Sakai	2012C-4227
chromosome-encoded									
*aidA*-I	-	-	2 (1)	3 (3)	2	2 (1)	1	1	-
*astA*	1	1	1	1	1	-	1	1	1
*bor* (*iss*)	1	1	1	-	2	1	1	2	1
*eae* type	β	β	γ	γ	β	ε	θ	γ	ε
*efa*1	2 (1)	2	(+/−)	1	1	2	1	(+/−)	1
*ehaA*	1	1	1	1	1	1	1	1	1
*espI*	1	1	1	-	-	1	-	-	1
*gad*	1	1	1	-	1	1	1	1	1
*iee*	(+/−)	(+/−)	1	1	1	1	1	1	-
*iha*	-	-	1	1	2	-	1	1	-
*lpf* cluster	1	1	1 (1)	1	2	1	2	2 (1)	1
*pagC*	-	-	-	1	-	2	1	1	-
*sod* (Cu/Zn)	1	1	2	2	-	2	2	2	1
*stx* genes	*stx*_2a_	*stx*_2a_	*stx*_2a_	*stx*_2a_	*stx*_1a_	*stx*_1a_ + *stx*_2a_	*stx*_1a_ + *stx*_2a_	*stx*_1a_ + *stx*_2a_	*stx*_1a_ + *stx*_2a_
*tir*	1	1	1	1	1	1	1	1	1
plasmid-encoded									
*cba*	1	1	-	-	-	-	-	-	-
*cma*	1	1	-	-	-	-	-	-	-
*ecf*	-	-	1	1	1	1	1	1	1
*ehxA* subtype	E	E	C	C	C	F	C	B	C
*espI*	1	1	-	-	-	-	-	-	-
*espP*	-	-	1	-	-	-	(+/−)	1	1
*katP*	-	-	-	-	1	-	-	1	1
*sfp*	1	1	-	-	-	-	-	-	1
*sta*1	1	1	-	-	-	-	-	-	-
*stcE*	-	-	-	1	-	1	-	1	-
*toxB*	-	-	1	-	1	-	-	1	-

present (≥ 90% identity, ≥ 80% gene coverage); −, absent; (+/−), partial (≥ 90% identity, 10–80% coverage);

numbers in parentheses indicate pseudogene(s)

Of the remaining two plasmids identified in O145:H25, one was a prophage plasmid similar to Enterobacteria phage P1 (GenBank: NC_005856.1) (99% nucleotide sequence identity with 80% sequence coverage) also found to be present in O165 and O111 (Table 1). The third plasmid present in both EHEC O145:H25 carried the *sfp* fimbrial gene cluster that was present on the pEHEC plasmids of O165 and the sorbitol-fermenting O157:HNM [17, 30, 31].

### Prophages and IS elements

**Fig. 3:**
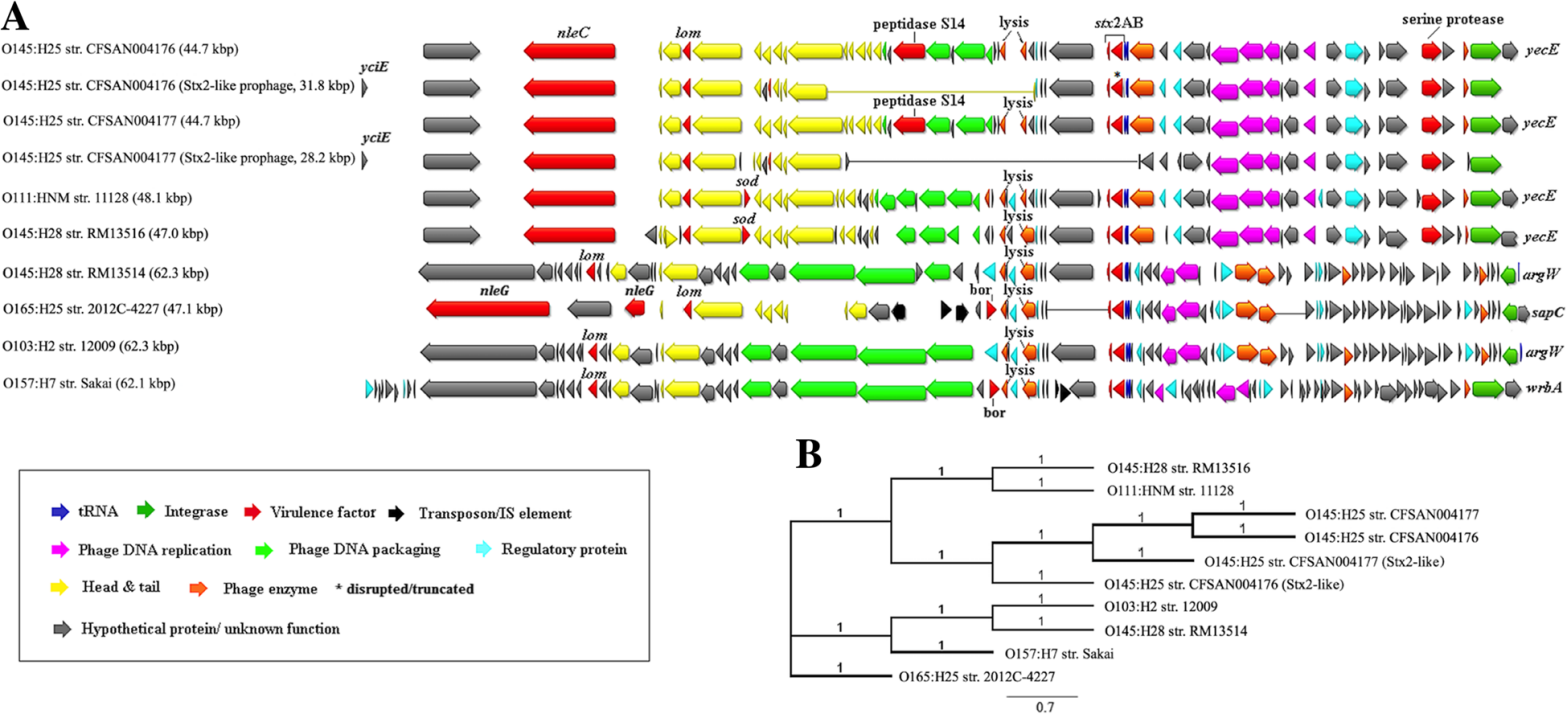
Genetic organization and phylogeny of the Stx_2a_ prophages identified in O145:H25 and other EHEC strains. **a** Integration sites are displayed on either sites and genes are colored based on functional characteristics as described in the figure legend. Approximate prophage sizes are indicated in parentheses. Nucleotide sequences were aligned in Geneious 9.1.5 using MAFFT with default settings. **b** Phylogenetic analysis of the Stx_2a_ prophages. The maximum-likelihood tree was constructed in Geneious 9.1.5 using RAxML and the GTR + GAMMA + Invariable sites model and 500 bootstrap replicates. The scale bar indicates the number of substitutions per base; branch support values 1 representing 100

MGEs are important factors in EHEC genome diversity and plasticity and play a major role in virulence evolution by means of HGT among and within bacterial species [14, 24, 25]. In fact, the most important virulence factors of all disease-associated STEC and EHEC strains, the Shiga toxin-encoding genes *stx*_1_ and *stx*_2_, are located on mobile bacteriophages. Overall, we identified 14 prophages in both O145:H25 strains, of which lambda-like prophages are predominant (Additional file 4: Table S2). Similarly, O145:H28 and other EHEC strains were found to carry 12 to 20 predominantly lambda-like prophages (Table 1) [14, 24]. Furthermore, the majority of the O145:H25 prophages were located near common integration sites and similar to previous findings were found to carry several non-LEE encoded effector (Additional file 4: Table S2 and Table S5) [14, 24]. Like O145:H28, both O145:H25 strains carry a prophage encoding the highly HUS-associated Stx_2a_ variant. However, while the two O145:H25 strains carry a closely related approximately 45 kbp lambda-like Stx_2__a_ prophage located adjacent to the *yecE* locus, O145:H28 (RM13514) was found to carry an approximately 60 kbp Podoviridae family Stx_2__a_ prophage inserted at *argW* [14]. Phylogenetic analysis suggests that the lambda-like Stx_2a_ prophages of O145:H25, O145:H28 (RM13516) and O111 are closely related to each other while being distantly related to those found in O145:H28 (RM13514) and the other EHEC strains (Fig. 3), indicating their lineage-independent acquisition. Interestingly, CFSAN004176 contains a second “Stx_2__a_-like” prophage (CFSAN004176_P01) with several nonsense mutations within the *stx*_2_A subunit and CFSAN004177 carries a closely related prophage (CFSAN004177_P01) at the same insertion site (*yciE*) without the *stx*_2_A and *stx*_2_B genes (Fig. 3). Both “Stx_2_-like” prophages appear to be defective as they are lacking genes primarily encoding DNA packaging-, phage tail- and lysis-associated proteins (Fig. 3).

IS elements are widely distributed among bacteria and play an important role in genome evolution and diversification [14, 24, 32]. They have further been proposed to contribute to improved bacterial fitness and adaptation to environmental changes due to their capability to modulate gene expression and induce insertional gene inactivation [33]. Overall, we identified 101 and 102 IS elements in the chromosomes of O145:H25, a considerably higher IS copy number as compared to O145:H28 and other EHEC strains (excluding O165) (Table 1). The majority of IS elements was found to be located on prophages, plasmids and IEs, supporting their possible implication in the fixation of MGEs in the bacterial genome [32]. Interestingly, we observed significant differences in the prevalence of certain IS elements among EHEC strains. For example, while IS629 was found to be the predominant IS among all EHEC strains with copy numbers ranging from 9 to 37 [14, 24] we identified only 3, non-intact IS629 in both O145:H25 strains. In contrast, O145:H25 carry over 50 copies of IS600 while the majority of the other EHEC strains carries less than 10 copies or zero (Additional file 4: Table S3). Moreover, most IS600 copies (45) appear to be active, as indicated by the presence of complete terminal repeats on both sides, suggesting that the evolution and diversification of O145:H25 was primarily driven by IS600-mediated mutations and not by IS629 as has been shown for O157 and suggested for other EHEC strains [14, 24, 32].

**Fig. 4:**
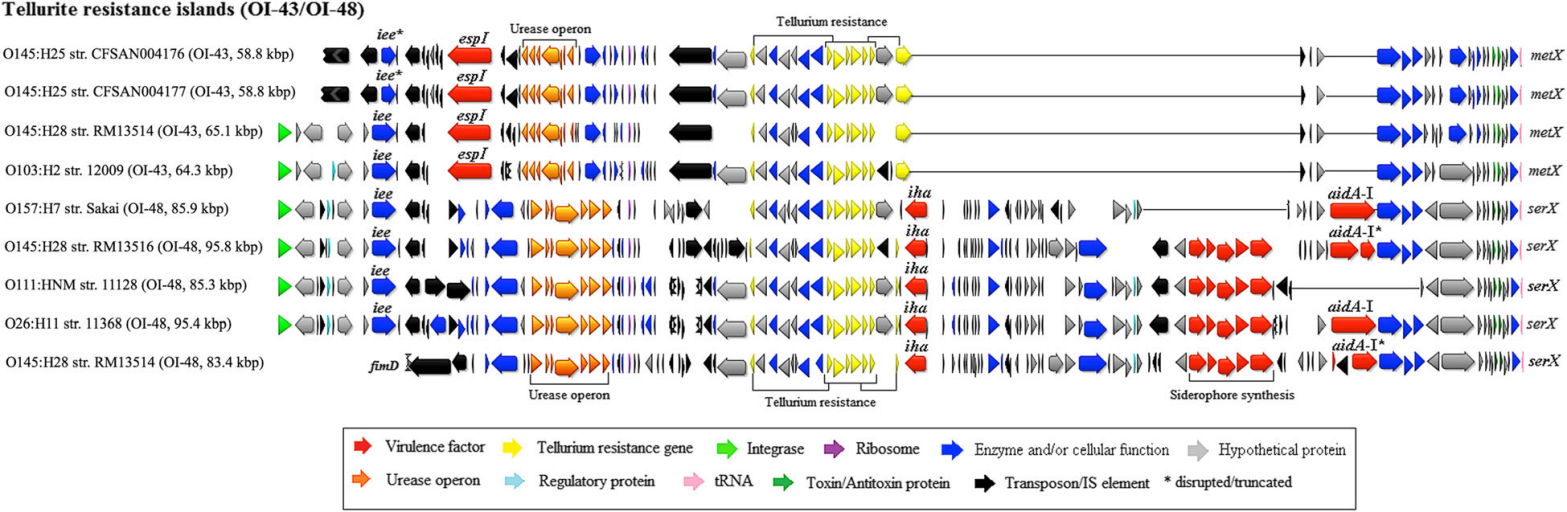
Genetic organization and content of the tellurite resistance islands OI-43/OI-48 identified in the EHEC strains. Approximate sizes of the OIs are indicated in parentheses. tRNA integration sites are displayed on the right and genes are colored based on functional characteristics as described in the figure legend. Nucleotide sequences were aligned in Geneious 9.1.5 using MAFFT with default settings

IS3 family members, such as IS629 and IS600, are believed to transpose in a replicative manner. While IS proliferation is generally an important factor in genome evolution as they can generate beneficial mutations that promote bacterial adaptation [34, 35], uncontrolled IS proliferation can be detrimental to the host. Thus, various genetic strategies have evolved to maintain stable IS copy numbers [33, 36, 37], one of which is the insertion sequence excision enhancer (IEE) that promotes the excision and deletion of IS3 family members [38]. While O145:H28, O157 Sakai, O26, O111 and O103 all carry the *iee* gene, in both O145:H25 strains *iee* is disrupted by IS600 while being completely absent in O165 (Fig. 4). This may explain the rather high copy number present in both O145:H25 strains and O165. However, in the presence of high copy numbers, negative regulators that inhibit or attenuate IS transpositions have been shown to be overexpressed [33, 36]. By implication, lowering the copy number (i.e. due to IEE-mediated IS deletion) increases IS transposition activity. Hence, the absence of *iee* accompanied by high IS copy numbers in O145:H25 and O165 suggests that IS transposition might be continuously depressed in those strains while being promoted in O145:H28 and the other EHEC strains. Furthermore, lower IS activity associated with decreased ability to change suggests that EHEC O145:H25 and O165 may not be able to adapt as rapidly to environmental changes as O145:H28 and other EHEC strains, which in turn might be one explanation why these serotypes are less commonly isolated [2, 13, 16]. Indeed, highly pathogenic and clinically relevant EHEC/STEC strains were found to carry *iee* and IS3 family members while less common EHEC/STEC strains were devoid of at least one of these elements [39].

### Genomic islands and Integrative elements

**Fig. 5:**
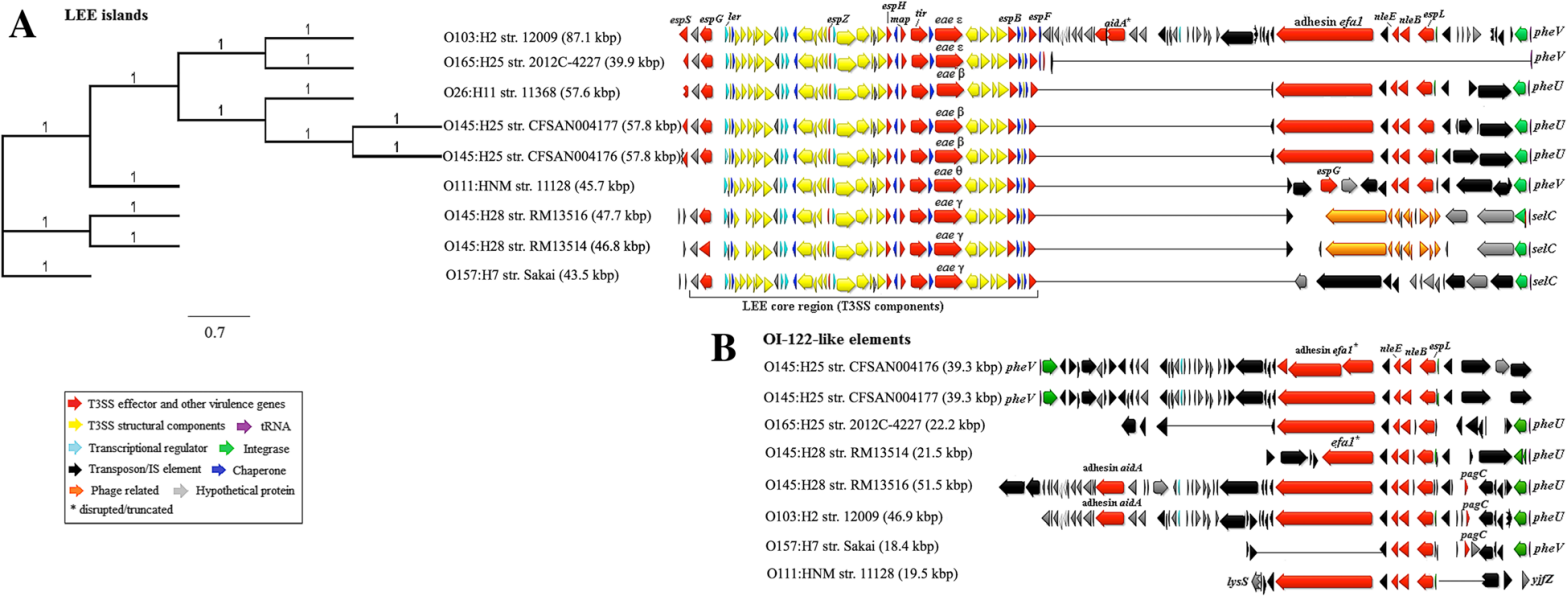
Genetic organization of the LEE islands and OI-122 identified in O145:H25 and other EHEC strains. tRNA integration sites are displayed on either sites and genes are colored based on functional characteristics as defined in the figure legend. Approximate sizes of the LEE and OI-122 are indicated in parentheses. Nucleotide sequences of the LEE and OI-122 were aligned in Geneious 9.1.5 using MAFFT with default settings. **a** Phylogeny and gene organization of the identified LEE islands. The maximum-likelihood tree was constructed in Geneious 9.1.5 using RAxML and the GTR + GAMMA + Invariable sites model and 500 bootstrap replicates. The scale bar indicates the number of substitutions per base; branch support values 1 representing 100. **b** Gene organization of OI-122 identified in the EHEC strains in comparison to the LEE-AR displayed above

Genomic islands occupy large chromosomal regions (> 10 kbp) and often carry genes providing selective advantages to the bacteria that carry them [25]. Based on their gene content, genomic islands are referred to metabolic, fitness and pathogenicity islands, the latter of which are absent in nonpathogenic bacteria. Several genomic islands have been identified in EDL933 [25], of which OI-28 (RTX-toxin), OI-35 (energy metabolism), OI-47 (adhesin and fatty-acid biosynthesis) and OI-138 (fatty acid biosynthesis) appear to separate the non-O157 EHEC from the O157 lineages (including EPEC O55 and EHEC O145:H28) as they are absent in O145:H25 and all other non-O157 EHEC strains while being present in O145:H28 (OI-47 is partially conserved in RM13514) (Additional file 3: Table S2). Other large OIs such as OI-115 (T3SS and *Salmonella*/*Shigella*-associated host-cell invasion genes) and OI-7 (T6SS) are partially conserved in O145:H25 and other EHEC strains while being absent in O145:H28 (lacks OI-7) and O165 (lacks both OIs) indicating potential evolutionary regression (Additional file 3: Table S2). The remaining large OIs, namely the PAIs OI-43 and OI-48 (tellurite resistance and urease gene cluster), OI-122 (toxins), and OI-148 (LEE) are present in all EHEC strains (O165 lacks OI-43/48) albeit with significant variations in size and gene contents (Figs. 4 and 5). In particular, the LEE islands of O145:H25 are approximately 58 kbp in size, are integrated at tRNA *pheU* and carry the intimin subtype beta. In contrast, the LEE islands of O145:H28 are about 10 kbp smaller, are integrated at the tRNA *selC* locus and carry the intimin subtype gamma, analogous to O157 [14]. Furthermore, while the LEE accessory regions (LEE-AR) of O145:H25 were found to contain the OI-122 encoding the virulence associated adhesin Efa1 and three T3SS effector proteins [3, 27, 40] the LEE-AR of O145:H28, O157, and O165 are devoid of any virulence factors but carry OI-122 outside the LEE (Fig. 5). Others proposed that the LEE and OI-122 were originally acquired as one large mosaic PAI and that in some strains, such as O157, the LEE and OI-122 separated later on [27]. However, both O145:H25 and other EHEC strains carry a second OI-122, suggesting that these two PAIs may indeed have separate origins [24, 28]. In fact, while the LEE core regions are well conserved among various EHEC strains the LEE-ARs are highly heterogeneous or unoccupied [14, 24] (Fig. 4). Thus, it appears likely that insertions (e.g. OI-122) were introduced into a preexisting LEE core and later on passed horizontally among different serotypes as exemplified by O145:H25 and O26 that carry a nearly identical LEE-OI-122 PAI (99% sequence identity). Indeed, phylogenetic analysis indicates that the LEEs of O145:H25 and O26 are highly related whereas the LEEs of O145:H28 are more closely related to the LEE in O157 (Fig. 5).

Of the two identical tellurite resistance islands (OI-43 and OI-48) identified in EDL933 [25] both O145:H25 strains were found to carry OI-43 that is highly similar to those found in O145:H28 (RM13514) and O103. However, RM13514 carries a second, much larger tellurite resistance island (OI-48) similar to those present in O145:H28 (RM13516), O26, O111 and O157 Sakai [14] (Fig. 4). In contrast to EDL933, significant differences were observed in the gene contents of these two islands. Specifically, while OI-43 encodes the putative virulence-associated serine protease EspI [41], OI-48 was found to carry genes encoding two adherence-associated proteins, Iha and AIDA-I, and a siderophore cluster involved in iron acquisition [42–44]. The presence of several IS elements within both tellurite resistance islands suggests that IS-mediated deletions/insertions played a role in the evolution of these PAIs (Fig. 4) and because closely related O165 was devoid of OI-43/48, these PAIs were acquired in a lineage-independent manner.

Noteworthy, besides the identified CRISPR/Cas system involved in phage immunity, both O145:H25 strains acquired what appear to be two distinct restriction-modification (RM) systems (identified by the presence of endonuclease and methylase genes) associated with phage-resistance (IE05 and IE07) (Fig. 1, Additional file 4: Table S2) [45]. Both of these IEs were absent in all EHEC genomes analyzed herein, in fact a BLASTN comparison against the NCBI database revealed that IE05 appears to be unique to O145 while IE07 was also present in O104:H7. Furthermore, IE05 was found to carry a potentially third phage-resistant system associated with abortive infection (Abi). In contrast to RM and CRISPR that prevent phage infections, Abi systems are triggered post phage infection and were shown to lead to bacterial cell death. However, whether the presence of these phage-resistant islands is associated with the acquisition of fewer prophages in O145:H25 as compared to the other EHEC genomes, as well as, the rarity of this particular serotype remains to be determined as many phages have been shown to be capable of evading phage-resistance systems [45].

### Adhesins and other colonization-contributing factors

Bacterial adhesion and their ability to persist in a host depends on a variety of different colonization-contributing factors (CCF). The LEE-encoded adhesin intimin (encoded by *eae*) and its translocated receptor Tir as well as several non-LEE-encoded effectors have been shown to play a key role for the intimate attachment of EHEC strains in the human and bovine gastrointestinal tract (GIT) [3, 26, 40]. However, several other genetic features are involved in this complex process including the plasmid-encoded EspP [46], ToxB [47], Ecf cluster [48] and StcE [49] as well as a number of fimbrial (i.e. Sfp and Lpf) [31, 40, 50, 51] and afimbrial adhesins (i.e. Iha, Efa1, AIDA-I and EhaA) [42, 52–54]. Similar to other EHEC serotypes, EHEC O145:H25 was found to carry *eae*, numerous non-LEE-encoded effectors (Additional file 4: Table S5), *efa*1, *ehaA*, and *lpf* but lacked *aidA*-I, *iha*, *espP*, *stcE* and *toxB,* many of which were equally absent in the other EHEC serotypes especially in O165 (Table 2). Furthermore, resistance genes such as *bor* [55], *sod* [56], *gad* [57], *katP* [58] and *pagC* [59] associated with serum survival and resistance towards oxidative stress were less prevalent in O145:H25 as compared to the other EHEC strains. (Table 2). More importantly, in contrast to all other EHEC strains analyzed herein, O145:H25 lacked the *ecf*-cluster associated with bacterial persistence in the bovine GIT which might explain why O145:H25 have thus far failed to be detected in any ruminant species. In fact, while *ecf*-positive strains were shown to persist in the bovine gut for up to 3 months *ecf*-deletion mutants were not recovered beyond seven days [48].

In addition, the acquisition of nutrients as well as other resources such as iron are equally important for the successful colonization of any given environment [44, 60]. Iron has been shown to be essential for bacterial growth and many EHEC strains including O145:H28, O26 and O157 were found to have acquired additional iron-acquisition systems such as siderophores or those located in OI-140 [14, 24, 25] (Fig. 4, Additional file 3: Table S2). In contrast, EHEC O145:H25 was devoid of any of these systems, indicating that they might be impaired in their ability to acquire iron in a stable microbiota, particularly during early stages of the colonization process. Thus, while O145:H25 generally appear to be able of colonizing the bovine gut, the absence of the *ecf*-cluster as well as additional iron acquisition systems suggests that they might be impaired in their ability to establish a persistent colonization.

Fimbriae are believed to mediate initial attachment of the bacteria to the epithelial cells and thus have been proposed to influence the tissue tropism observed in O157 and other EHEC strains [51, 61, 62]. Overall, we identified 15 apparently intact fimbrial gene cluster in O145:H25 a similar range as compared to other EHEC strains (Table 3). Interestingly, while many fimbrial gene cluster were shared among all EHEC strains others appear to be lineage-specific. In particular, whereas clusters F03, F04, F10 and F13 were present in all EHEC strains of the non-O157 lineages, in O145:H28 and O157 these clusters were either absent or they carried non-identical homologs instead (Table 3, Additional file 3: Table S3). Similarly, cluster F08 (OI-1) was present in closely related O157 and O145:H28 while being only partially conserved in the other EHEC strains. Furthermore, all EHEC strains were found to carry *lpf*-clusters however, while the majority of the non-O157 EHECs carried the *lpf*-cluster initially identified in O113 [50], O157 carried *lpf*_OI-141_ and *lpf*_OI-154_ while O145:H28 carried *lpf*_OI-141_. The observed differences among the fimbrial gene cluster present in the various EHEC serotypes may indicate adaptation to distinct ecological niches. In fact, it was previously shown that O157 primarily colonizes the lowest portion of the small intestine whereas other EHEC strains such as O26 and O111 were found to interact primarily with the large intestinal mucosa [63–66]. The observed tissue tropism was further proposed to be influenced by the intimin type. Hence, since the fimbrial profile and intimin type of O145:H25 are more similar to that of O26 and O111, and O145:H28 are more similar to O157 (Tables 2 and 3), O145:H25 and O145:H28 likely adapted to different ecological niches within the mammalian host.

**Table 3:** Fimbrial gene clusters present in EHEC O145:H25 and other *E. coli* strains

Fimbrial cluster	O145:H25		O145:H28		O26:H11	O103:H2	O111:HNM	O157:H7	O165:H25	commensal
	CFSAN004176	CFSAN004177	RM13514	RM13516	11,365	12,009	11,128	Sakai	2012C-4227	HS
F01	+	+	+	+	+	+	+	H	+	+
F02	+	+	H^a^	H^a^	H^b^	+	H^b^	H^c^	H^b^	H^b^
F03	(+)	(+)	−	−	+	+	(+)	−	(+)	+
F04	+	+	−	−	(+)	+	+	−	+	+
F05 (OI-127)	+	+	−	−	+	+	+	(+)	+	+
F06 (Lpf_O113/OI-154)	+	+	−	−	+	−	+	H	+	−
F07	+	+	+	+	+	+	+	+	+	+
F08 (OI-1)	(+/−)	(+/−)	(+)	(+)	(+/−)	(+/−)	(+/−)	(+)	(+/−)	(+/−)
F09	+	+	+	+	+	+	+	+	+	+
F10	+	+	H^d^	H^d^	+	+	+	H^d^	(+)	(+/−)
F11	+	(+)	+	+	+	+	−	+	−	(+/−)
F12	+	+	+	+	+	+	+	+	+	+
F13	+	+	H^e^	H^e^	+	(+)	+	H^e^	+	+
F14	+	+	+	+	+	(+)	+	(+)	(+)	+
F15	+	+	+	(+)	+	+	+	+	(+)	(+)
F16 (OI-61)	(+/−)	(+/−)	+	(+/−)	+	(+/−)	+	+	(+/−)	(+/−)
F17 (OI-141)	−	−	(+)	+	H^f^	H^f^	H^f^	(+)	−	−
F18 (OI-47)	−	−	(+/−)	(+/−)	−	−	−	+	−	−
F19 (*sfp* cluster)	+	+	−	−	−	−	−	−	+	−
Total^g^	15 (2)	15 (2)	13 (1)	12 (2)	16 (1)	14 (2)	15 (1)	16	14 (2)	11 (4)

+, present (> 90% identity, > 90% gene coverage); −, absent; (+/−), partial; (+), contains pseudogene(s); H homolog (< 90% sequence identity); (see also Additional file 3: Table S3)

^a, b, c, d, e, f^same letters indicate same homolog

^g^numbers in parentheses indicate the number of partially conserved clusters

## Conclusion

Phylogenetic analysis supported by a comprehensive comparative genomic scrutiny revealed that EHEC O145:H25 and O145:H28 evolved from two different clonal lineages. The lineage-independent acquisition of various MGEs as well as gene loss contributed to the divergence of these pathogens also reflected in variations seen in their virulence repertoire. Furthermore, based on the identified CCF profile, ruminants such as cattle could be a natural reservoir for EHEC O145:H25 as has been shown for other EHEC strains. However, the absence of several genetic features such as the *ecf*-cluster and additional iron-acquisition systems present in other EHEC strains suggests that EHEC O145:H25 might be impaired in their ability to establish a persistent colonization. This might explain why these strains have not been detected in any ruminant species. In addition, marked variations in fimbrial gene clusters and intimin subtypes between EHEC O145:H25 and O145:H28 suggests a potential adaptation to different colonization sites within the mammalian host’s GIT.

## Methods

### Bacterial Strains

EHEC O145:H25 strains CFSAN004176 and CFSAN004177 were isolated from patients in the USA that developed HUS in 2003 and 2004, respectively. Both strains were obtained from the Centers for Disease Control and Prevention (Atlanta, GA).

### Genome sequencing and assembly

Bacteria were grown in tryptic soy broth as previously described [17]. Bacterial DNA was extracted from 2 mL of bacterial cell culture using the automated QIAcube extraction system (Qiagen Inc., Valencia, CA) according to the manufacturer’s instructions for Gram-negative bacteria.

Sequencing was carried out using single molecule real time (SMRT) DNA sequencing on the Pacific Bioscience *RS* II Sequencer (PacBio, Menlo Park, CA) as previously described [17]. Briefly, template DNA was sheared to ≥10-kbp using g-TUBEs (Covaris, Inc., Woburn, MA). Whole genome libraries were prepared according to the PacBio 10-kbp insert library protocol using DNA Template Kit 1.0 and afterwards size-selected using the BluePippin size-selection system (Sage Science, Inc., Beverly, MA) according to the manufacturer’s instruction. Libraries were sequenced using P4/C2 Chemistry Kits on five SMRT cells with a 180-min collection protocol. The obtained sequence reads were analyzed by SMRT Analysis 2.3.0 (PacBio, Menlo Park, CA) and *de novo* assembled using the PacBio hierarchical genome assembly process 3 (HGAP3.0)/Quiver software package.

### Gap closure, genome annotation and accession numbers

The resulting assemblies for each strain were confirmed by optical maps generated with 30 fold coverage on the Argus Mapping Station (OpGen, Gaithersburg, MD) as previously described [67]. Briefly, high molecular weight DNA was immobilized onto derivatized glass surfaces, digested with BamHI restriction enzyme (OpGen), and fluorescently stained with YOYO-1 dye (OpGen). The fragments were then size measured using an automated fluorescent microscope (OpGen). Each sequence contig was aligned to the optical map using MapSolver Software version 3.1 (OpGen) in order to identify contig order, overlapping contigs, potential mis-assemblies and inversions [68]. Overlapping contigs were combined by removing the overlapping, identical sequence of one contig. The resulting, combined contig was verified by mapping all PacBio raw sequencing reads back to this contig using SMRT Analysis. Finally, contig closure was determined by creating dot plots for each contig utilizing the Gepard software as previously described [17].

Genome sequences were annotated by the NCBI Prokaryotic Genome Automatic Annotation Pipeline (PGAAP) (http://www.ncbi.nlm.nih.gov/genome/annotation_prok/) and are available at GenBank under the following accession numbers: CFSAN004176 chromosome (NZ_CP014583), pCFSAN004176_EHEC (NZ_CP012493), pCFSAN004176_sfp (NZ_CP012492), pCFSAN004176_PP (NZ_CP012491), CFSAN004177 chromosome (CP014670), pCFSAN004177_EHEC (CP012495), pCFSAN004177_sfp (CP012496), pCFSAN004177_PP (CP012494).

### Comparative genomics and phylogenetic analysis

In order to identify genomic dissimilarities among O145:H25 and other EHEC strains BLAST Ring Image Generator (BRIG) [69] was used for comparison and visualization purposes. EHEC O145:H25 strain CFSAN004177 served as a reference genome and the nucleotide sequences of the following EHEC chromosomes were BLASTed against the reference strain with default settings: O145:H25 strain CFSAN004176, O165:H25 strain 2012C-4227 (GenBank: CP013029.1), O145:H28 strain RM13514 (GenBank: NZ_CP006027.1), O145:H28 strain RM13516 (GenBank: NZ_CP006262.1), O157:H7 strain Sakai (GenBank: NC_002695.1), O26:H11 strain 11,365 (GenBank: NC_013361.1), O103:H2 strain 12,009 (GenBank: NC_013353.1), and O111:HNM strain 11,128 (GenBank: NC_013364.1). Non-homologous regions as identified by the BRIG image were further manually inspected using CLC Genomics Workbench 7.5 (CLC bio, Boston, MA).

The phylogenetic relatedness of the newly sequenced O145:H25 EHEC strains among 67 fully sequenced *E. coli* strains (Additional file 1: Table S1) was determined by cgMLST analysis using Ridom SeqSphere + 2.4.0 as previously described [70]. First a cgMLST scheme was defined by using the Ridom software’s target definer tool with default settings and *E. coli* O157:H7 Sakai (GenBank: NC_002695.1) as reference genome (5498,450 bases, 5204 genes). To establish a list of core and accessory genome genes the following six fully sequenced genomes were used for comparison with the reference genome: *E. coli* O157:H7 strain EC4115, *E. coli* O157:H7 strain EDL933, *E. coli* O157:H7 strain TW14359, *E. coli* O55:H7 strain CB9615, *E. coli* O55:H7 strain RM12579, and *E. coli* O157:H7 strain Xuzhou21 (Additional file 1: Table S1). Multiple gene copies identified in any of the seven genomes were removed from the analysis as failed genes (553 targets). Then a task template was created containing both core and accessory genes for future analysis. Each individual locus of the identified core (3860 targets) and accessory genes (791 targets) was assigned allele number 1. Afterwards, each individual *E. coli* genome was queried against the created task template and all identified loci that were 100% identical to the reference and query genomes were assigned allele number 1; otherwise a new allele number was called for that locus. The number of shared loci among the 69 genomes was determined to be 1371 loci (using Sakai genome as reference). The cgMLST performed a gene-by-gene analysis of each individual genome for the 69 strains, and SNP’s found within the identified 1371 shared alleles were used to determine their genetic distance (Additional file 2: Dataset S1 and S2). The phylogenetic analysis involved 69 nucleotide sequences and a total dataset of 86,350 SNP’s positions with all ambiguous positions being removed for each sequence pair. The SNP-based phylogenetic tree was constructed in MEGA 6 [71] using the Neighbour-joining algorithm [72] with the evolutionary history and evolutionary distance inferred using the Minimum Evolution method [73] and the Kimura 2-parameter model [74], respectively.

### *In silico* MLST analysis, serotyping and determination of virulence and antimicrobial resistance genes

*In silico* MLST analysis was performed based on the seven housekeeping genes (*adk*, *fumC*, *gyrB*, *icd*, *mdh*, *purA* and *recA*) previously described for *E. coli* [75]. Allelic variants of those seven gene loci were identified using Ridom SeqSphere + 2.4.0 (Ridom GmbH, Münster, Germany) and alleles numbers and STs were assigned according to the *E. coli* MLST database (http://mlst.warwick.ac.uk/mlst/dbs/Ecoli). Briefly, each genome sequence was screened against all in the MLST database available alleles for the seven loci. Once a 100% sequence match was identified an allele number was assigned to that loci. STs were only assigned if the obtained allele combination for the seven loci was already described in the MLST database otherwise a new ST was called.

Serotypes were confirmed *in silico* using Ridom software by screening the obtained genome sequences for the presence of O-type (*wzx* and *wzy*) and H-type genes (*fliC*) as previously described [76, 77]. Genome sequences were further screened for all virulence [78] and antimicrobial resistance genes [79] deposited in the Center for Genomic Epidemiology database (http://www.genomicepidemiology.org/) using Ridom for performing batch screening of the genomes analyzed as described elsewhere [76]. Briefly, all the genes were divided into classes or groups by homology in fasta format (e.g. all *ast*A alleles were in a single fasta file), and used as a task template. Afterwards a project was created using all these task templates, and each WGS was screened for the presence of each gene class (virulence or antimicrobial gene). Ninety-five virulence genes and 14 antimicrobial classes were analyzed by this method. The threshold used for screening for these genes using the Ridom software was > 90% identity and > 80% aligning or gene coverage in order to be called as present. When a pseudogene is present, Ridom visualize it as a red gene, when present and complete is visualized as green, and when absent the name appears as gray out.

### Identification and comparative analysis of prophages, IEs and IS elements

Prophages and prophage-like elements of the newly sequenced O145:H25 EHEC strains were initially identified using the prophage-predicting PHASTER web server [80]. IEs such as pathogenicity and genomic islands were analyzed using the genomic island prediction web server IslandViewer3 for initial identification [81]. Each of the identified prophages, prophage-like elements and IEs were then manually inspected for accuracy using CLC Genomics Workbench 7.5 by locating nearby integrases and identification of potential integration sites. In order to identify sequence similarity and synteny among the identified prophages and IEs of the newly sequenced O145:H25 strains and other EHEC strains, prophage and IE sequences were aligned, analyzed and visualized using MAFFT Alignment with default settings in Geneious 9.1.5 (Biomatters, Auckland, New Zealand). Phylogenetic analysis was inferred using RAxML with the GTR + GAMMA + Invariable sites model and assessed by 500 bootstrap replicates using Geneious 9.1.5.

IS elements were initially identified using ISfinder [82]. Copy number and presence of each identified IS element were confirmed by BLASTing the nucleotide sequences of each IS element against the genome sequences of the nine EHEC genomes using BLASTN. Nucleotide sequences with at least 90% identity and coverage to the matching IS element were considered a match, coverage between 10% and 90% were considered a partial match. Each IS element was further BLASTed against all identified IS elements of the nine EHEC genomes to identify highly similar sequences among different IS elements. Highly similar IS sequences were BLASTed against the EHEC genomes simultaneously and the BLASTN results compared regarding sequence location and identity in order to avoid multiple matches for different IS elements at the same position. Of note, notably fewer IS were identified in all EHEC strains as compared to previous results, mainly attributable to different search parameters used herein. For example, previously identified IS elements such as IS1414 or IS630 shared less than 90% sequence identity in all EHEC strains and thus were not considered a match. Other copy numbers of certain IS could not be confirmed regardless of which parameters were applied (particularly in both O145:H28 strains).

### Availability of supporting data

The genome sequences of CFSAN004176 and CFSAN004177 were deposited in GenBank and are available under the following links:

- CFSAN004176 chromosome https://www.ncbi.nlm.nih.gov/nuccore/NZ_CP014583.1;
- pCFSAN004176_EHEC https://www.ncbi.nlm.nih.gov/nuccore/NZ_CP012493.1;
- pCFSAN004176_sfp https://www.ncbi.nlm.nih.gov/nuccore/NZ_CP012492.1;
- pCFSAN004176_PP https://www.ncbi.nlm.nih.gov/nuccore/NZ_CP012491.1;
- CFSAN004177 chromosome https://www.ncbi.nlm.nih.gov/nuccore/CP014670.1;
- pCFSAN004177_EHEC https://www.ncbi.nlm.nih.gov/nuccore/CP012495.1;
- pCFSAN004177_sfp https://www.ncbi.nlm.nih.gov/nuccore/CP012496.1;
- pCFSAN004177_PP https://www.ncbi.nlm.nih.gov/nuccore/CP012494.1.

All other supporting data are included in this published article and its supplementary information files.

## Additional files

Additional file 1: Table S1. Metadata and accession numbers of the 69 *E. coli* strains used for phylogenetic analysis. (XLSX 17 kb)

Additional file 2: **Dataset S1**. SNP’s and SNP positions identified in the 1371 gene loci shared among the 69 *E. coli* genomes. **Dataset S2**. cgMLST for each of the 1371 identified gene loci shared among the 69 *E. coli* genomes. (XLSX 23678 kb)

Additional file 3: Table S1. Genomic dissimilarities between O145:H25 and other EHEC genomes. **Table S2.** Conservation of the EDL933-specific OIs in O145:H25 and the other EHEC strains. **Table S3.** Fimbrial gene cluster identified in the nine EHEC genomes, including nucleotide sequence identity. (XLSX 65 kb)

Additional file 4: Table S1. *In silico* MLST analysis of the nine EHEC strains**. Table S2.** Identified prophages and IEs in O145:H25. **Table S3.** IS elements present on EHEC chromosomes. **Table S4.** IS elements present on EHEC plasmids. **Table S5.** LEE- and non-LEE-encoded effectors present on EHEC chromosomes. (DOCX 59 kb)

## Abbreviations

CCF: Colonization-contributing factor
CDS: Coding DNA sequence
cgMLST: Core genome multi locus sequence typing
EHEC: Enterohemorrhagic *Escherichia coli*
GIT: Gastrointestinal tract
HC: Hemorrhagic colitis
HGT: Horizontal gene transfer
HUS: Hemolytic uremic syndrome
IE: Integrative element
IEE: Insertion sequence excision enhancer
IS: Insertion sequence
LEE: Locus of enterocyte effacement
MGE: Mobile genetic element
OI: O island
PAI: Pathogenicity island
SNP: Single nucleotide polymorphism
STEC: Shiga toxin producing *Escherichia coli*
