# Supplementary material for: Genome sequencing and comparative genomics of enterohemorrhagic Escherichia coli O145:H25 and O145:H28 reveal distinct evolutionary paths and marked variations in traits associated with virulence & colonization
Source: BMC Microbiol. 2017 Aug 22;17:183. doi: 10.1186/s12866-017-1094-3 (PMC5567499; doi:10.1186/s12866-017-1094-3)
Supplement: Supplementary file 4 — In silico MLST analysis of the nine EHEC strains. Table S2. Identified prophages and IEs in O145:H25. Table S3. IS elements present on EHEC chromosomes. Table S4. IS elements present on EHEC plasmids. Table S5. LEE- and non-LEE-encoded effectors present on EHEC chromosomes. (DOCX 59 kb) [file 12866_2017_1094_MOESM4_ESM.docx]

**Additional File 4**

**Table S1: *In silico* MLST analysis of the nine EHEC strains.**

| **strain** | **serotype** | **identified allele numbers of the seven *E. coli* MLST gene loci** | | | | | | | **ST** | **ST Cplx** |
| --- | --- | --- | --- | --- | --- | --- | --- | --- | --- | --- |
|  |  | ***adk*** | ***fumC*** | ***gyrB*** | ***icd*** | ***mdh*** | ***purA*** | ***recA*** |  |  |
| CFSAN004176 | O145:H25 | 437 | 46 | 69 | 1 | 600 | 34 | 50 | 7061 | none |
| CFSAN004177 | O145:H25 | 437 | 46 | 69 | 1 | 600 | 34 | 50 | 7061 | none |
| RM13514 | O145:H28 | 19 | 23 | 18 | 24 | 21 | 2 | 16 | 32 | 32 |
| RM13516 | O145:H28 | 19 | 23 | 51 | 24 | 21 | 5 | 16 | 6130 | none |
| 2012C-4227 | O165:H25 | 48 | 46 | 43 | 45 | 11 | 34 | 35 | 119 | none |
| 11368 | O26:H11 | 16 | 4 | 12 | 16 | 9 | 7 | 7 | 21 | 29 |
| 11128 | O111:HNM | 6 | 4 | 12 | 16 | 9 | 7 | 12 | 16 | 29 |
| 12009 | O103:H2 | 6 | 4 | 3 | 17 | 7 | 7 | 6 | 17 | 20 |
| Sakai | O157:H7 | 12 | 12 | 8 | 12 | 15 | 2 | 2 | 11 | 11 |

**Table S2: Identified prophages and integrative elements (IE) in CFSAN004176 and CFSAN004177**

| **Name** | **Position (start)** | **Position (end)** | **Size (bp)** | **GC %** | **Insertion site** | **Type** | **Virulence related genes and other genetic features** |
| --- | --- | --- | --- | --- | --- | --- | --- |
| **EcO145:H25 str. CFSAN004176**  **Prophages** | | |  |  |  |  |  |
| CFSAN004176_P01 | 1  5173938 | 12040  5193735 | 31838 | 51.4 | *yciE* | Enterobacteria phage phiP27 (lambda-like) | serine protease, tRNA's (Arg, Met), *stx*_2a_^*^, *lom*, hydrolase, *nleC* |
| CFSAN004176_P02 | 290193 | 352626 | 62434 | 49.6 | *ynfE* | Enterobacteria phage BP-4795 (lambda-like) | *espS*, *lom**, *terB*, tRNA's (Arg, Met), *nleG* |
| CFSAN004176_P03 | 644162 | 689684 | 45523 | 52.2 | *yecE* | Enterobacteria phage phiP27 (lambda-like) | *nleC*, *lom*, peptidase S14, *stx*_2a_, serine protease |
| CFSAN004176_P04 | 778634 | 813872 | 35239 | 48.8 | *potC* | Enterobacteria phage BP-4795 (lambda-like) | *terB*, *espX*-homolog, *nleC* |
| CFSAN004176_P05 | 962333 | 1009592 | 47260 | 52.2 | *yccA* | Enterobacteria phage lambda | *terB*, *espV*, tRNA's (Arg, Met) |
| CFSAN004176_P06 | 1234340 | 1253347 | 19008 | 47.0 | *ybhB* | Stx2 converting phage vB_EcoP_24B (Podoviridae-like) | *traR*, *nleG*, *espJ* |
| CFSAN004176_P07 | 2068148 | 2120241 | 52094 | 48.3 | *prfC* | Enterobacteria phage mEp460 (lambda-like) | *fosA* |
| CFSAN004176_P08 | 2516812 | 2531434 | 14623 | 47.2 | *yjbN* | Enterobacteria phage mEp460 (lambda-like) | *nleG*, *nleG*, *lom* |
| CFSAN004176_P09 | 4450762 | 4484194 | 33433 | 44.9 | tRNA (*argW*) | Enterobacteria phage Sf6 (P22-like) |  |
| CFSAN004176_P10 | 4513462 | 4547782 | 34321 | 52.6 | *yhdJ* | Enterobacteria phage P88 (*Myoviridae*) |  |
| CFSAN004176_P11 | 4824278 | 4841873 | 17596 | 45.1 | *yegL* | Enterobacteria phage BP-4795 (lambda-like) | *nleH*, *espJ*, *cif* |
| CFSAN004176_P12 |  | 4993162 | 38204 | 50.5 | tRNA (*serU*) | Enterobacteria phage YYZ-2008 (unclassified) | *sod*, *lom*, *nleG*, *nleG*, *espW*-homolog, *nleG* |
| CFSAN004176_P13 | 5006317 | 5029338 | 23022 | 47.2 | *icD* | Enterobacteria phage HK630 (lambda-like) | *traR*, *bor*, *lom**, *hydrolase*, *ompT* |
| CFSAN004176_P14 | 5131193 | 5162151 | 30595 | 53.0 | *ompW* | Enterobacteria phage BP-4795 (lambda-like) | *terB*, tRNA's (Arg, Met), colonization factor AcfC |
| **Integrative elements** |  |  |  |  |  |  |  |
| CFSAN004176_IE01 | 1723156 | 1748429 | 25273 | 48.2 | tRNA (*thrW*) |  | intimin-like adhesin *fdeC*, fimbrial operon |
| CFSAN004176_IE02 | 2187844 | 2200625 | 12782 | 48.1 | *yjjG* |  | restriction endonuclease |
| CFSAN004176_IE03 | 2205511 | 2222474 | 16964 | 48.0 | tRNA (*leuX*) |  | RM system, peptidase S8 |
| CFSAN004176_IE04 | 2363880 | 2421668 | 57789 | 40.2 | tRNA (*pheU*) | LEE | T3SS machinery, *espS*, *espG*, *espH*, *map*, *tir*, *eae*, *espA*, *espB*, *espD*, *espF*, *espZ*, adhesin *efa*1, *nleE*, *nleB*, *espL* |
| CFSAN004176_IE05 | 2971719 | 2987525 | 15807 | 44.9 | tRNA (*selC*) |  | putative two-component abortive infection system (Abi), RM system |
| CFSAN004176_IE06 | 3591528 | 3599538 | 8011 | 42.6 | *viuB*-*fimD* |  |  |
| CFSAN004176_IE07 | 3627041 | 3685885 | 58845 | 49.3 | tRNA (*metX*) | OI-43 | urease operon, tellurium resistance, *espI*, *iee** |
| CFSAN004176_IE08 | 3779169 | 3818453 | 39285 | 46.1 | tRNA (*pheV*) | OI-122 | *espL*, *nleB*, *nleE*, adhesin *efa*1* |
| CFSAN004176_IE09 | 5164611 | 5173058 | 8448 | 41.3 |  |  | *nleG*, *nleG*, *espO*, *nleF*, *nleH*, *espM* |
| **EcO145:H25 str. CFSAN004177**  **Prophages** | | |  |  |  |  |  |
| CFSAN004177_P01 | 1  5177474 | 14298  5191331 | 28156 | 51.4 | *yciE* | Enterobacteria phage phiP27 (lambda-like) | serine protease, hydrolase, *lom*, *nleC* |
| CFSAN004177_P02 | 26084 | 57048 | 30965 | 53.0 | *ompW* | Enterobacteria phage BP-4795 (lambda-like) | *terB*, tRNA's (Arg, Met), colonization factor AcfC |
| CFSAN004177_P03 | 158898 | 181917 | 23020 | 47.2 | *icD* | Enterobacteria phage HK630 (lambda-like) | *traR*, *bor*, *lom**, hydrolase, *ompT* |
| CFSAN004177_P04 | 195072 | 233276 | 38205 | 50.5 | tRNA (*serU*) | Enterobacteria phage YYZ-2008 (unclassified) | *sod*, *lom*, *nleG*, *nleG*, *espW*-homolog, *nleG* |
| CFSAN004177_P05 | 346360 | 363952 | 17593 | 45.1 | *yegL* | Enterobacteria phage BP-4795 (lambda-like) | *nleH*, *espJ*, *cif* |
| CFSAN004177_P06 | 640434 | 674748 | 34315 | 52.6 | *yhdJ* | Enterobacteria phage P88 (*Myoviridae*) |  |
| CFSAN004177_P07 | 704024 | 737453 | 33429 | 44.9 | tRNA (*argW*) | Enterobacteria phage Sf6 (P22-like) |  |
| CFSAN004177_P08 | 2659259 | 2672552 | 13294 | 47.2 | *yjbN* | Enterobacteria phage mEp460 (lambda-like) | *nleG*, *nleG*, *lom* |
| CFSAN004177_P09 | 3069067 | 3121150 | 52084 | 48.3 | *prfC* | Enterobacteria phage mEp460 (lambda-like) | *fosA* |
| CFSAN004177_P10 | 3935914 | 3955334 | 19421 | 47.0 | *ybhB* | Stx2 converting phage vB_EcoP_24B (Podoviridae-like) | *traR*, *nleG*, *espJ* |
| CFSAN004177_P11 | 4180028 | 4227274 | 47246 | 52.2 | *yccA* | Enterobacteria phage lambda | tRNA's (Met, Arg), *terB*, hydrolase, *espV* |
| CFSAN004177_P12 | 4375697 | 4410920 | 35224 | 48.8 | *potC* | Enterobacteria phage BP-4795 (lambda-like) | *terB*, *espX*-homolog, *nleC* |
| CFSAN004177_P13 | 4500642 | 4545386 | 44745 | 52.2 | *yecE* | Enterobacteria phage phiP27 (lambda-like) | *nleC*, *lom*, peptidase S14, *stx*_2a_, serine protease, tRNA's (Arg, Met) |
| CFSAN004177_P14 | 4836908 | 4899337 | 62430 | 49.6 | *ynfE* | Enterobacteria phage BP-4795 (lambda-like) | tRNA's (Arg, Met), *terB*, *lom**, *espS*, *nleG* |
| **Integrative elements** |  |  |  |  |  |  |  |
| CFSAN004177_IE01 | 15178 | 23624 | 8447 | 41.3 |  |  | *nleG*, *nleG*, *espO*, *nleF*, *nleH*, *espM* |
| CFSAN004177_IE02 | 1370992 | 1410271 | 39280 | 46.1 | tRNA (*pheV*) | OI-122 | *espL*, *nleB*, *nleE*, adhesin *efa*1 |
| CFSAN004177_IE03 | 1503548 | 1562383 | 58836 | 49.3 | tRNA (*metX*) | OI-43 | urease operon, tellurium resistance, *espI*, *iee** |
| CFSAN004177_IE04 | 1589882 | 1597892 | 8011 | 42.6 | *viuB*-*fimD* |  |  |
| CFSAN004177_IE05 | 2201868 | 2217673 | 15805 | 44.9 | tRNA (*selC*) |  | putative two-component abortive infection system (Abi), RM system |
| CFSAN004177_IE06 | 2767681 | 2825461 | 57781 | 40.1 | tRNA (*pheU*) | LEE | T3SS machinery, *espS*, *espG*, *espH*, *map*, *tir*, *eae*, *espA*, *espB*, *espD*, *espF*, *espZ*, adhesin *efa*1, *nleE*, *nleB*, *espL* |
| CFSAN004177_IE07 | 2966855 | 2983818 | 16964 | 48.0 | tRNA (*leuX*) |  | RM system*, peptidase S8 |
| CFSAN004177_IE08 | 2988704 | 3001483 | 12780 | 48.1 | *yjjG* |  | restriction endonuclease |
| CFSAN004177_IE09 | 3440858 | 3466130 | 25273 | 48.2 | tRNA (*thrW*) |  | intimin-like adhesin *fdeC*, fimbrial operon |

^*^ disrupted

**Table S3: Comparison of IS elements present on EHEC chromosomes using BLASTN (coverage ≥ 90%, identity ≥ 90%)**

| **Strains** | **IS family** | **size (bp)** | **O145:H25** | |  | **O145:H28** | | **O26:H11** | **O103:H2** | **O111:HNM** | **O157:H7** | **O165:H25** |
| --- | --- | --- | --- | --- | --- | --- | --- | --- | --- | --- | --- | --- |
|  |  |  | **CFSAN004176** | **CFSAN004177** |  | **RM13514** | **RM13516** | **11365** | **12009** | **11128** | **Sakai** | **2012C-4227** |
| IS1H | IS1 | 764 | 1 | 1 |  | 0 | 0 | 2 (1) | 2 (1) | 2 (1) | 2 (1) | 1 |
| IS1F | IS1 | 768 | 0 | 0 |  | 2 | 4 | 4 | 1 | 2 | 1 | 1 |
| IS2 | IS3 | 1331 | 3 (3) | 3 (3) |  | 3 (3) | 1 | 1 (1) | 2 (1) | 3 (2) | 1 | 29 (5) |
| IS3 | IS3 | 1258 | 0 | 0 |  | 0 | 0 | 4 (2) | 1 | 1 | 0 | 8 |
| **IS600** | **IS3** | **1264** | **53 (6)** | **54 (5)** |  | **9 (1)** | **2 (2)** | **8 (2)** | **0** | **1 (1)** | **2 (2)** | **22 (8)** |
| **IS629** | **IS3** | **1310** | **3 (3)** | **3 (3)** |  | **31 (2)** | **19 (6)** | **9 (3)** | **28 (5)** | **37 (3)** | **20 (4)** | **31 (2)** |
| IS911 | IS3 | 1250 | 4 | 4 |  | 1 | 1 (1) | 1 | 0 | 1 (1) | 0 | 0 |
| ISEc16 | IS3 | 1244 | 1 (1) | 1 (1) |  | 0 | 2 (2) | 1 | 2 (2) | 0 | 1 (1) | 0 |
| ISEc31 | IS3 | 1258 | 0 | 0 |  | 0 | 0 | 0 | 0 | 0 | 2 (1) | 0 |
| ISEc48 | IS3 | 1336 | 1 | 1 |  | 0 | 0 | 0 | 0 | 0 | 1 (1) | 0 |
| ISCfr6 | IS3 | 1258 | 2 | 2 |  | 1 | 0 | 0 | 1 | 4 | 0 | 0 |
| ISEc13 | IS4 | 1550 | 3 (3) | 3 (3) |  | 1 (1) | 2 (2) | 1 (1) | 4 (4) | 4 (4) | 2 (2) | 0 |
| ISCro3 | IS4 | 1430 | 0 | 0 |  | 1 (1) | 0 | 1 (1) | 0 | 1 (1) | 1 (1) | 0 |
| IS30 | IS30 | 1221 | 0 | 0 |  | 0 | 3 (3) | 2 (2) | 0 | 2 (2) | 4 (3) | 0 |
| IS682 | IS66 | 2533 | 0 | 0 |  | 1 | 1 | 1 | 0 | 1 | 1 | 0 |
| ISEc8 | IS66 | 2442 | 4 (2) | 4 (2) |  | 2 (1) | 6 (1) | 5 (3) | 3 (2) | 3 (3) | 11 (3) | 0 |
| ISEc22 | IS66 | 2454 | 0 | 0 |  | 0 | 0 | 7 (1) | 0 | 0 | 0 | 0 |
| ISEc23 | IS66 | 2532 | 14 (8) | 14 (8) |  | 2 (2) | 8 (2) | 1 (1) | 5 (5) | 1 | 1 (1) | 0 |
| ISEc47 | IS66 | 2541 | 0 | 0 |  | 1 (1) | 0 | 0 | 2 (2) | 0 | 0 | 1 (1) |
| ISEc49 | IS66 | 2766 | 0 | 0 |  | 1 (1) | 0 | 0 | 0 | 0 | 0 | 0 |
| ISCro1 | IS66 | 2699 | 0 | 0 |  | 0 | 0 | 9 | 1 | 0 | 0 | 0 |
| ISSfl3 | IS66 | 2729 | 1 (1) | 1 (1) |  | 0 | 1 (1) | 1 (1) | 2 (2) | 1 (1) | 1 (1) | 0 |
| IS91 | IS91 | 1829 | 0 | 0 |  | 0 | 0 | 0 | 0 | 2 | 0 | 0 |
| IS621 | IS110 | 1425 | 0 | 0 |  | 0 | 0 | 13 | 12 | 2 (2) | 0 | 0 |
| ISEc20 | IS110 | 1459 | 0 | 0 |  | 7 | 6 | 0 | 1 | 1 | 0 | 0 |
| IS1414 | IS256 | 1314 | 0 | 0 |  | 1 (1) | 0 | 1 (1) | 0 | 1 (1) | 0 | 0 |
| ISSoEn2 | IS256 | 1315 | 0 | 0 |  | 1 (1) | 1 (1) | 0 | 0 | 2 (2) | 1 (1) | 0 |
| ISEc18 | IS481 | 1020 | 0 | 0 |  | 1 (1) | 0 | 1 (1) | 0 | 1 (1) | 1 (1) | 0 |
| ISEc38 | ISL3 | 1722 | 3 (1) | 3 (1) |  | 1 | 1 | 2 | 1 | 0 | 0 | 0 |
| IS609 | IS200/IS605 | 1748 | 1 | 1 |  | 2 | 2 | 3 | 2 | 5 (2) | 2 | 1 |
| ISEc1 | ISAs1 | 1291 | 7 (2) | 7 (2) |  | 1 (1) | 1 (1) | 5 (1) | 5 (2) | 5 (1) | 4 | 6 (3) |
| ISEc26 | ISAs1 | 1305 | 0 | 0 |  | 0 | 0 | 1 | 1 | 1 | 1 | 0 |
| **Total** |  |  | 101 (30) | 102 (29) |  | 70 (17) | 61 (22) | 84 (22) | 76 (26) | 84 (28) | 60 (23) | 100 (19) |

Partial IS elements are indicated in parentheses (coverage ≥ 10% - < 90% with ≥ 90% identity)

**Table S4: Comparison of IS elements present on EHEC plasmids using BLASTN (coverage ≥ 90%, identity ≥ 90%)**

| **Strains** | **IS family** | **size (bp)** | **O145:H25** | |  | **O145:H28** | | **O26:H11** | **O103:H2** | **O111:HNM** | **O157:H7** | **O165:H25** |
| --- | --- | --- | --- | --- | --- | --- | --- | --- | --- | --- | --- | --- |
|  |  |  | **CFSAN004176** | **CFSAN004177** |  | **RM13514** | **RM13516** | **11365** | **12009** | **11128** | **Sakai** | **2012C-4227** |
| **pEHEC** |  |  |  |  |  |  |  |  |  |  |  |  |
| IS1H | IS1 | 764 | 0 | 0 |  | 0 | 0 | 0 | 0 | 0 | 1 (1) | 0 |
| IS3 | IS3 | 1258 | 0 | 0 |  | 0 | 0 | 0 | 0 | 0 | 2 (2) | 0 |
| ISEc17 | IS3 | 1258 | 0 | 0 |  | 2 (2) | 0 | 2 (2) | 0 | 1 (1) | 0 | 3 (1) |
| IS600 | IS3 | 1264 | 1 | 1 |  | 4 (3) | 0 | 2 | 0 | 0 | 1 (1) | 2 (1) |
| IS629 | IS3 | 1310 | 1 (1) | 1 (1) |  | 4 (2) | 3 (2) | 3 (2) | 6 | 6 (3) | 3 (2) | 4 (2) |
| IS911 | IS3 | 1250 | 0 | 0 |  | 1 | 1 (1) | 1 | 2 (2) | 0 | 1 | 0 |
| ISCfr6 | IS3 | 1258 | 0 | 0 |  | 0 | 2 | 0 | 0 | 0 | 0 | 0 |
| IS679 | IS66 | 2704 | 1 (1) | 1 (1) |  | 0 | 0 | 0 | 0 | 0 | 0 | 0 |
| ISEc8 | IS66 | 2442 | 0 | 0 |  | 2 (2) | 2 | 2 (2) | 1 (1) | 3 (1) | 0 | 0 |
| ISEc23 | IS66 | 2532 | 1 (1) | 1 (1) |  | 0 | 1 | 0 | 0 | 0 | 0 | 0 |
| ISEc47 | IS66 | 2541 | 0 | 0 |  | 1 (1) | 0 | 1 (1) | 0 | 0 | 0 | 0 |
| ISSfl3 | IS66 | 2729 | 0 | 0 |  | 3 (3) | 0 | 3 (3) | 0 | 0 | 0 | 0 |
| IS91 | IS91 | 1829 | 2 (1) | 2 (1) |  | 3 (2) | 4 | 3 (1) | 0 | 3 | 1 (1) | 2 |
| IS1294 | IS91 | 1689 | 1 (1) | 0 |  | 0 | 0 | 0 | 0 | 0 | 0 | 0 |
| ISEc37 | IS91 | 1828 | 0 | 0 |  | 1 (1) | 0 | 1 (1) | 0 | 0 | 1 (1) | 0 |
| ISSbo1 | IS91 | 1709 | 3 (3) | 3 (2) |  | 0 | 0 | 0 | 0 | 0 | 0 | 0 |
| ISSm1 | IS110 | 1369 | 1 | 1 |  | 0 | 0 | 0 | 0 | 0 | 0 | 0 |
| ISEc38 | ISL3 | 1722 | 0 | 0 |  | 2 (2) | 0 | 2 (2) | 0 | 0 | 0 | 1 (1) |
| **other plasmids** |  |  |  |  |  |  |  |  |  |  |  |  |
| IS1R | IS1 | 768 | 0 | 0 |  | 0 | 0 | 0 | NA | 4 | 0 | 0 |
| IS600 | IS3 | 1264 | 2 | 2 |  | 0 | 0 | 0 | NA | 0 | 0 | 0 |
| IS629 | IS3 | 1264 | 0 | 0 |  | 2 | 0 | 0 | NA | 1 | 0 | 0 |
| IS1203 | IS3 | 1310 | 0 | 0 |  | 0 | 0 | 0 | NA | 5 | 0 | 0 |
| ISEc25 | IS3 | 1310 | 1 | 1 |  | 0 | 0 | 0 | NA | 0 | 0 | 0 |
| IS10L | IS4 | 1329 | 0 | 0 |  | 0 | 0 | 0 | NA | 1 | 0 | 0 |
| IS26 | IS6 | 820 | 0 | 0 |  | 3 | 0 | 0 | NA | 4 | 0 | 0 |
| IS100 | IS21 | 1953 | 1 | 1 |  | 0 | 0 | 0 | NA | 0 | 0 | 0 |
| ISEc23 | IS66 | 2532 | 2 (1) | 2 (1) |  | 0 | 0 | 0 | NA | 0 | 0 | 0 |
| IS1294 | IS91 | 1689 | 1 (1) | 1 (1) |  | 0 | 0 | 1 | NA | 0 | 0 | 0 |
| ISSbo1 | IS91 | 1709 | 1 | 1 |  | 0 | 0 | 1 (1) | NA | 0 | 0 | 0 |
| ISVsa3 | IS91 | 977 | 0 | 0 |  | 1 | 0 | 0 | NA | 0 | 0 | 0 |
| ISEc38 | ISL3 | 1722 | 1 (1) | 1 (1) |  | 0 | 0 | 0 | NA | 0 | 0 | 0 |
| IS609 | IS200/IS605 | 1748 | 0 | 0 |  | 0 | 1 (1) | 0 | NA | 0 | 0 | 0 |
| **Total** |  |  | 20 (11) | 19 (9) |  | 29 (18) | 14 (4) | 22 (15) | 9 (3) | 28 (5) | 10 (8) | 12 (5) |

Partial IS elements are indicated in parentheses (coverage ≥ 10% - < 90% with ≥ 90% identity)

**Table S5: LEE- and non-LEE-encoded effectors present on EHEC chromosomes**

| **Strains** | **O145:H25** | |  | **O145:H28** | | **O26:H11** | **O103:H2** | **O111:HNM** | **O157:H7** | **O165:H25** |
| --- | --- | --- | --- | --- | --- | --- | --- | --- | --- | --- |
|  | **CFSAN004176** | **CFSAN004177** |  | **RM13514** | **RM13516** | **11365** | **12009** | **11128** | **Sakai** | **2012C-4227** |
| *espA* (variant) | 1 (β) | 1 (β) |  | 1 (γ) | 1 (γ) | 1 (β) | 1 (β) | 1 (α) | 1 (γ) | 1 (β) |
| *espB* (variant) | 1 (β) | 1 (β) |  | 1 (γ) | 1 (γ) | 1 (β) | 1 (β) | 1 (α) | 1 (γ) | 1 (β) |
| *espD* (variant) | 1 (β) | 1 (β) |  | 1 (γ) | 1 (γ) | 1 (β) | 1 (β) | 1 (α) | 1 (γ) | 1 (β) |
| *espF* | 1 | 1 |  | 1 | 1 | 1 | 1 | 1 | 1 | 1 |
| *espG* | 1 | 1 |  | 1 | 1 | 1 | 1 | 1 | 1 | 1 |
| *espH* | 1 | 1 |  | 1 | 1 | 1 | 1 | 1 | 1 | 1 |
| *espJ* | 2 | 2 |  | 1 | 1 | 1 | 0 | 1 | 1 | 1 |
| *espK* | 1 | 1 |  | 1 | 1 | 2 | 2 | 1 | 1 | 2 |
| *espL* | 2 | 2 |  | 1 | 1 | 1 | 2 | 2 | 1 | 1 |
| *espM* | 1 | 1 |  | 1 | 1 | 1 | 1 | 1 | 1 | 1 |
| *espN* | 1 | 1 |  | 1 | 1 | 1 | 1 | 1 | 1 | 1 |
| *espO* | 1 | 1 |  | 2 | 2 | 2 | 1 | 2 | 2 | 2 |
| *espR* | 1 | 1 |  | 1 | 1 | 1 | 1 | 1 | 1 | 1 |
| *espS* | 2 | 2 |  | 2 | 2 | 2 (1) | 2 | 1 | 0 | 2 (1) |
| *espV* | 1 (1) | 1 (1) |  | 2 (1) | 2 (1) | 1 (1) | 1 (1) | 1 (1) | 1 (1) | 1 (1) |
| *espW* | 1 | 1 |  | 0 | 0 | 1 | 1 | 1 | 1 | 1 |
| *espX* | 2 | 2 |  | 1 | 1 | 1 | 1 | 1 | 1 | 1 |
| *espZ* (*sepZ*) | 1 | 1 |  | 1 | 1 | 1 | 1 | 1 | 1 | 0 |
| *map* | 1 | 1 |  | 1 | 1 | 1 | 1 | 1 | 1 | 1 |
| *nleA* | 1 | 1 |  | 1 | 1 | 2 | 1 | 1 | 1 | 1 |
| *nleB* | 2 | 2 |  | 3 (1) | 3 (1) | 1 | 4 | 3 (1) | 3 (1) | 2 (1) |
| *nleC* | 3 | 3 |  | 2 (1) | 3 (1) | 1 | 2 (2) | 2 | 1 | 2 |
| *nleD* | 0 | 0 |  | 0 | 0 | 0 | 0 | 0 | 1 | 1 |
| *nleE* | 2 | 2 |  | 1 | 1 | 1 | 2 | 2 | 1 | 1 |
| *nleF* | 1 | 1 |  | 1 | 1 | 1 | 1 | (1) | 1 | 1 |
| *nleG* | 9 | 9 |  | 5 | 5 | 13 | 7 (1) | 10 | 13 (2) | 9 |
| *nleH* | 2 | 2 |  | 3 (1) | 2 | 2 | 2 | 2 | 2 | 2 |
| *tccp* | 1 | 1 |  | 1 | 1 | 1 | 1 | 1 | 1 (1) | 1 (1) |
| *tir* (variant) | 1 (β) | 1 (β) |  | 1 (γ) | 1 (γ) | 1 (β) | 1 (β) | 1 (α) | 1 (γ) | 1 (β) |
| *cif* | 1 (1) | 1 (1) |  | 1 | 1 | 1 (1) | 1 (1) | 1 (1) | 0 | 0 |
| **Total** | 46 (2) | 46 (2) |  | 40 (4) | 40 (3) | 46 (3) | 43 (5) | 45 (4) | 45 (5) | 43 (4) |

numbers in parentheses indicate the number of pseudogenes
